# Ambient air pollution exposure and full-term birth weight in California

**DOI:** 10.1186/1476-069X-9-44

**Published:** 2010-07-28

**Authors:** Rachel Morello-Frosch, Bill M Jesdale, James L Sadd, Manuel Pastor

**Affiliations:** 1 Department of Environmental Science, Policy and Management, University of California, Berkeley, 137 Mulford Hall, Berkeley CA 94720-3114, USA; 2 School of Public Health, University of California, Berkeley, 137 Mulford Hall, Berkeley CA 94720-3114, USA; 3 Department of Environmental Sciences, Occidental College, 1600 Campus Road, Los Angeles, CA 90041, USA; 4 Program on Environmental and Regional Equity, University of Southern California, 3620 S. Vermont Ave, KAP-462, Los Angeles, CA 90089-0255, USA; 5 Department of American Studies and Ethnicity, University of Southern California, 3620 S. Vermont Ave, KAP-462, Los Angeles, CA 90089-0255, USA

## Abstract

**Background:**

Studies have identified relationships between air pollution and birth weight, but have been inconsistent in identifying individual pollutants inversely associated with birth weight or elucidating susceptibility of the fetus by trimester of exposure. We examined effects of prenatal ambient pollution exposure on average birth weight and risk of low birth weight in full-term births.

**Methods:**

We estimated average ambient air pollutant concentrations throughout pregnancy in the neighborhoods of women who delivered term singleton live births between 1996 and 2006 in California. We adjusted effect estimates of air pollutants on birth weight for infant characteristics, maternal characteristics, neighborhood socioeconomic factors, and year and season of birth.

**Results:**

3,545,177 singleton births had monitoring for at least one air pollutant within a 10 km radius of the tract or ZIP Code of the mother's residence. In multivariate models, pollutants were associated with decreased birth weight; -5.4 grams (95% confidence interval -6.8 g, -4.1 g) per ppm carbon monoxide, -9.0 g (-9.6 g, -8.4 g) per pphm nitrogen dioxide, -5.7 g (-6.6 g, -4.9 g) per pphm ozone, -7.7 g (-7.9 g, -6.6 g) per 10 *μ*g/m^3 ^particulate matter under 10 μm, -12.8 g (-14.3 g, -11.3 g) per 10 *μ*g/m^3 ^particulate matter under 2.5 μm, and -9.3 g (-10.7 g, -7.9 g) per 10 *μ*g/m^3 ^of coarse particulate matter. With the exception of carbon monoxide, estimates were largely unchanged after controlling for co-pollutants. Effect estimates for the third trimester largely reflect the results seen from full pregnancy exposure estimates; greater variation in results is seen in effect estimates specific to the first and second trimesters.

**Conclusions:**

This study indicates that maternal exposure to ambient air pollution results in modestly lower infant birth weight. A small decline in birth weight is unlikely to have clinical relevance for individual infants, and there is debate about whether a small shift in the population distribution of birth weight has broader health implications. However, the ubiquity of air pollution exposures, the responsiveness of pollutant levels to regulation, and the fact that the highest pollution levels in California are lower than those regularly experienced in other countries suggest that precautionary efforts to reduce pollutants may be beneficial for infant health from a population perspective.

## Background

Nearly 6.2% of all singleton births in the U.S. are low birth weight infants [1]. Low birth weight (LBW) is an important predictor of infant mortality and future child health status[2-4], including risk of cardiovascular disease [5,6] and cognitive development [7,8]. Indeed, the fetal origins hypothesis posits that *in utero *delays in growth and development can increase the risk of many chronic diseases throughout the life course [9]. A strong body of literature has shed much light on the individual-level risk factors (e.g., health behaviors, inter-pregnancy interval, socioeconomic status, race/ethnicity, and access to adequate health care) [10-14] as well as place-based factors (e.g. social inequality, neighborhood quality and support networks) [15-18] that are associated with low birth weight.

In the past decade, an increasing number of studies within the United States and elsewhere have identified a relationship between air pollution and birth weight. These studies primarily focus on the commonly monitored air pollutants, including ozone (O_3_), particulate matter (PM_2.5_, PM_10_), carbon monoxide (CO), nitrogen oxides (NO_2 _or NO_X_), and sulfur dioxide (SO_2_). Results from these studies are inconsistent in terms of singling out a particular pollutant that is consistently inversely associated with birth weight or elucidating potential windows of susceptibility of the fetus by trimester of exposure. Some of these studies have examined air pollution effects on birth weight measured continuously as well as categorically (e.g. <2500 grams). Several reviews have examined the evidence linking air pollution and LBW [19-24], although inconsistencies in study design have precluded a systemic meta-analysis of the literature. Despite difficulties in synthesizing the literature, reviews have generally concluded that the body of evidence suggests small effects of air pollution exposure on birth weight and that additional investigation is needed to better understand which pollutants and which trimester of exposure appear to cause adverse effects in the fetus.

Air pollution is hypothesized to affect the fetus directly through transplacental exposure or indirectly by adversely impacting maternal health during pregnancy [19]. With the exception of CO which is known to cross the placental barrier and bind efficiently with fetal hemoglobin, the mechanism of toxicity of air pollution on the fetus is poorly understood [25,26]. Although toxicity mechanisms remain unclear, several have been proposed, particularly for PM effects, including oxidative stress, pulmonary and placental inflammation, blood coagulation, endothelial dysfunction and changes in diastolic and systolic blood pressure [27].

California has been the focus of many air quality and birth outcome studies, in part because of its persistent ambient air quality problems. Studies in Southern California have found positive associations between last trimester exposure to CO and particulate matter less than 10 μm in aerodynamic diameter (PM_10_) and full-term low birth weight [26,28]. Two additional California studies found LBW associations for PM_2.5 _but not CO when examining births throughout the entire state [29] and for O_3 _and CO for births during 1975-1987 in several Southern California cities [30]. A study in Massachusetts and Connecticut found that an inter-quartile increase in gestational exposure to NO_2_, CO, PM_10 _and PM_2.5 _lowered birth weight, and that effect estimates for PM_2.5 _were higher for African American versus White mothers [31]. A national study linked term births to average county-level PM exposures for 2001-2003 and found that results varied markedly by region, with strong associations in the Northwest versus null associations in the Southwest. After controlling for region, the small positive association between PM exposure and LBW in multivariate models lost statistical significance [32]. Internationally, results have also been mixed. Studies in Brazil, Australia and Germany found positive associations between exposure to PM and LBW [33-35], while studies in Canada and Taiwan found null or weak associations [36,37]. Other studies found small associations with exposures to other pollutants such as CO, NO_2_, and SO_2_, and LBW [33,34,36,38,39].

Different results across studies may be due to differences in how studies control for confounders, regional and national variations in underlying health conditions among populations, differences in pollution measurement techniques, spatial and temporal differences in exposure assessment, composition of the pollutants examined (e.g. PM composition and size), study sample size, and statistical modeling techniques [19,21,23,24]. Although the effects of air pollution on birth weight appear to be small, current findings have important implications for infant health due to the ubiquity of exposures to many of the air pollutants within and outside the United States. Moreover, evidence suggests that certain socio-demographic groups may be more vulnerable to the adverse effects of air pollution on infant health [31,40], although this issue has not been extensively examined.

This study builds upon existing work by analyzing the effect of air pollution on average birth weight and risk of low birth weight in California. We used California and federal monitoring data for PM_2.5_, PM_10_, CO, NO_2_, SO_2_, and ozone, to assess the relationship between ambient air pollution exposures and birth weight among infants born between 37-44 weeks gestation during the years 1996-2006. We also estimated ambient exposures to coarse PM, where coarse particle exposure was defined as the difference in ambient exposures for respirable and fine particles (PM_10 _- PM_2.5_).

## Methods

We calculated pollutant exposures during pregnancy using monitoring data from all monitors within a specified radius of the census tract or ZIP Code of the mother's residence. For each birth, we calculated averages for the time periods corresponding to the 9 months of pregnancy as well as for each trimester; trimester-specific exposures were examined to identify potentially critical times during pregnancy when pollutants may affect birth weight. We assessed effects for birth weight, measured continuously and categorically. We also analyzed the potential confounding and interaction effects of individual-level and contextual-level measures of socioeconomic status based on previous work [31,32].

### Natality Data

Data for this analysis came from several sources that were merged using spatial and temporal variables. We acquired tract and ZIP Code geocoded birth data from the California Department of Health Services Natality files for 1996-2006 (California Automated Vital Statistics System, 2006, unpublished data). Of the 5,905,277 birth records in these files, 5,886,969 were among California residents. California reports locations of maternal residence at both census tract and ZIP Code levels. As a proxy for neighborhood of residence, we assigned births reported with a valid 2000 census tract to that tract code. Remaining births reported with a valid 1990 census tract were assigned that tract code. If neither a valid 2000 tract code nor a valid 1990 tract code was reported on the birth record, then a ZIP Code matching a valid census 2000 ZIP Code Tabulation Area (ZCTA) was used as the relevant geocode for the birth. Census tracts are designed to contain a relatively homogenous population of a few thousand residents, though there can be great variability with respect to geographic area and population. ZCTAs are organized by the postal service for the delivery of mail, and tend to be somewhat larger than tracts, at least in urbanized areas [41]. 5,835,930 births could be assigned a valid tract or ZCTA location by these methods.

We restricted our analysis to singleton live births (5,670,630), with a gestational age between 37-44 weeks (4,888,421) with a known birth weight (4,888,397), sex (4,888,374), date of birth (4,888,374), maternal educational attainment (4,801,979), parity (4,801,190), and a maternal age of 9 to 49 years old (4,800,679). Infants with a reported birth weight that is implausible for gestational age at delivery were excluded from all analyses using the method of Alexander et al. [42]. For example, among full-term births, those with a birth weight of 1,000 grams or less were excluded, as were those with a birth weight greater than 6,000 grams. This resulted in a potentially eligible sample size of 4,776,090, of whom 3,545,177 lived in a census tract or ZCTA at the time of delivery which was within 10 km of an air monitor in nearly continuous operation throughout the pregnancy.

Low birth weight was defined for infants delivered full-term as a birth weight of less than 2,500 grams, compared to a birth weight of 2,500 grams or more. Because maternal demographics are independently associated with birth weight [4,10,16,17] and air pollution [43], we added the following measures of maternal characteristics to our multivariate models: maternal age (9-14, 15-19, 20-34, 35-49 years old), educational attainment (<=6th grade, 7th - 11th grade, high school diploma or GED, 1-3 years of college, or >=4 years of college), maternal race/ethnicity (non-Hispanic White, Hispanic, non-Hispanic Black, non-Hispanic Indian/Alaska Native, non-Hispanic Asian or Pacific Islander, and non-Hispanic Other or Multiple Race), maternal birthplace (Mexico, other or unspecified foreign country, and United States). We also controlled for temporal variables, including calendar year and season of delivery (Jan-March, April-June, July-Sept, Oct-Dec), marital status, parity, Kotelchuk index of prenatal care adequacy (no prenatal care, inadequate, less than adequate, adequate, or unknown) [44], and presence of any vs. none of the following pregnancy risk factors: anemia, diabetes, chronic or pregnancy-associated hypertension, and/or herpes).

We also included four measures of neighborhood socio-economic status, measured cross-sectionally at the time of the 2000 census [41]. These measures included: neighborhood poverty rate- calculated as the proportion of residents living in households with an income under the federal poverty level (30% and higher, 20% to 29%, 10% to 19%, 5% to 9%, under 5%); neighborhood unemployment rate- calculated as the proportion of residents aged 16 years and older in the labor force who were currently looking for work (15% and higher, 10 - 14%, 7.5-10%, 5-7.5%, under 5%); home ownership- calculated as the proportion of households owned by their residents (under 20%, 20% to 39%, 40% to 59%, 60% to 79%, 80% and higher); neighborhood educational attainment rate, a measure of human capital that was calculated as the proportion of residents aged 25 and older with at least a high school education (20% and higher, 15% to 19%, 10% to 14%, 5% to 9%, under 5%). Values for 2000 census tracts and 2000 ZCTAs were calculated from the SF3 file of the 2000 census. Values for 1990 tracts were calculated using the Census Tract Relationship File to apportion 2000 population characteristics to 1990 tract geographic boundaries [45].

### Exposure Assessment

Information on the ambient concentrations of air pollutants came from two sources, the Environmental Protection Agency's Air Quality System (AQS) [46] and the California Aerometric Information Reporting System (CalAIRS) [47]. Concentration measurements for gaseous pollutants (CO, NO_2_, ozone and SO_2_) were usually reported in ppm and particulate air pollutants (PM_10_, PM_2.5_, and coarse PM) were usually reported in *μ*g/m^3^. Concentrations for these pollutants reported in other units (such as ppb) were transformed into the above units. The latitude and longitude of the monitor locations as reported in CalAIRS or AQS were validated by comparing the reported coordinates to address geocoding in Google Earth [Version 4.2.0205.5730, 2007].

Daily values of gaseous pollutants (CO, NO_2_, O_3 _and SO_2_) were calculated by averaging hourly measures, if there were at least 18 hourly measures in a day. Although gaseous pollutants were usually monitored daily, PM was less frequently measured, usually every three to six days. Particulate matter measures were usually reported as daily summaries. When they were not, daily averages of hourly measures were calculated, provided that there were at least 18 hourly measures in a day. If there was at least one valid daily summary of any gaseous or particulate pollutant in a week, a weekly summary for that pollutant was calculated by averaging the daily summaries in that week. Weekly air pollution concentration summaries were assigned to each tract and ZCTA by measuring the distance between the latitude and longitude of the active monitoring site closest to each census block centroid, while accounting for the curvature of the Earth. Block level weekly pollution estimates and distances for each pollutant were then averaged up to the tract and ZCTA levels using the population living within each block as a weighting factor.

Gestational age was reported in the natality file based on the mother's last menstrual period. We used this information to calculate air pollution exposure for each birth and pollutant for the entire pregnancy and each trimester. For each birth, full pregnancy and trimester-specific exposure measures were calculated by assigning each week of pregnancy the weekly average concentration measure for each pollutant specific to its geocode type (2000 or 1990 census tract, or 2000 ZCTA). Monthly summaries were then calculated by averaging the weekly summaries within each four week period after the last menstrual period. If there were fewer than three weekly summaries in a given month, it was not assigned a monthly summary concentration. First trimester summaries were calculated by averaging the first four monthly concentration averages, if none were missing. Second trimester summaries were calculated by averaging the 5th to 7th monthly averages, if none was missing. Third trimester summaries were calculated in like manner, depending on the number of weeks before delivery. Full pregnancy summaries were calculated by averaging all exposure estimates during pregnancy. We assigned a distance to each pregnancy with a valid pollutant exposure average using the maximum distance to an active monitor during any single week of pregnancy.

### Analysis

We used linear multivariable models (Statistical Analysis Software 9.2) to estimate the impact of air pollutants on birth weight as a continuous measure, and logistic regression models to estimate air pollution effects on birth weight as dichotomous outcome (<2500 grams versus ≥2500 grams). For PM, we estimated the birth weight effect in grams for each 10 *μ*g/m^3 ^increase in exposure; for CO, the measure was grams of birth weight per ppm; for O_3 _and NO_2_, the measure was grams of birth weight per part per hundred million (pphm); and for SO_2_, the measure estimated was grams of birth weight per ppb.

In addition to infant sex and gestation age, the maternal factors described above (maternal age, marital status, educational attainment, race/ethnicity, parity, maternal birthplace, prenatal care access, and presence of pregnancy risk factors) along with calendar year, season of delivery and area-level measures (neighborhood educational attainment, poverty rate, unemployment rate, and home ownership) were included in the multivariable models to obtain adjusted estimates. We ran logistic and linear models to examine trimester-specific effects on birth weight as well as effects from full-term pregnancy exposures. We also examined pollution effects on birth weight within strata of maternal race/ethnicity and neighborhood-level poverty rate to assess potential effect modification. Finally, we ran models with two pollutants included simultaneously to assess potential confounding effects of co-pollutants.

We estimated the effect of exposures limited to the population within a set of distance radii: 3 km, 5 km, and 10 km from monitors to assess whether effect estimates were sensitive to monitor distance from the mother's residential census tract or ZCTA. Thus, the number of births included at a longer radius includes those also assessed at a shorter radius.

## Results

Pollutant exposures were estimated for 3,545,177 singleton births, although not all births had available monitoring data for all pollutants. 2.3% of births included in the study were under 2,500 grams. Table 1 provides descriptive statistics comparing the characteristics of eligible singleton births and the study sample, consisting of births with a maternal residence within 10 km of an active monitor throughout pregnancy. Mothers in the study population were predominantly Hispanic or White, over half were born in the United States, and 59% of mothers included in the study had low educational attainment (completed high school or less). The study sample did not appear to differ appreciably from all eligible births. Full pregnancy pollutant exposure means and interquartile ranges are shown in Table 2. Correlation between gestational exposure estimates ranged from -55% between O_3 _and CO to 87% between coarse PM and PM_10_. Correlation with an absolute level above 70% consisted of: PM_2.5 _exposures had 72% and 74% correlation with NO_2 _and PM_10_, respectively; coarse PM had 87% correlation with PM_10_, and CO had 79% correlation with NO_2 _(data not shown). Pollutant levels averaged over the course of the pregnancy varied slightly by year and season of birth (data not shown).

**Table 1:** Characteristics of singleton births in study sample compared with overall population of singleton births at 37-44 weeks of gestational age, California (1996-2006).

	Total Eligible Singleton Births (n = 4,776,090)	Study Sample (n = 3,545,177)
low birth weight (<2,500 grams)	2.3%	2.3%
		
maternal age (years)		
9 to 14	0.1%	0.2%
15 to 19	9.9%	10.2%
20 to 34	74.2%	74.3%
35 to 49	15.8%	15.4%
		
educational attainment		
none to 11th grade	30.2%	31.5%
12th grade	27.6%	27.6%
1-3 years college	19.8%	19.4%
4+ years college	22.4%	21.4%
		
marital status		
married	42.8%	42.0%
not married	22.5%	23.7%
not on form	27.9%	27.4%
missing	6.8%	6.9%
		
maternal race/ethnicity		
Hispanic	49.6%	51.5%
Black (non-Hispanic)	5.8%	6.3%
American Indian/Alaska Native (non-Hispanic)	0.4%	0.3%
Asian Pacific Islander (non-Hispanic)	11.9%	12.0%
Other Race (non-Hispanic)	0.0%	0.0%
White (non-Hispanic)	32.2%	29.6%
missing	0.1%	0.1%
		
maternal birthplace		
Mexico	27.6%	28.6%
other or unknown foreign country	18.5%	19.2%
US and her territories	53.8%	52.1%
missing	0.1%	0.1%
		
parity		
first live birth	39.5%	39.7%
		
maternal risk factors		
anemia, diabetes, hypertension and/or herpes	4.5%	4.4%
none of the above	86.1%	86.4%
missing	9.4%	9.2%
		
Kotelchuk index		
no prenatal care	1.6%	1.8%
inadequate	9.0%	9.0%
intermediate	11.8%	11.7%
adequate	44.1%	43.8%
more than adequate	33.5%	33.7%
insufficient information	0.1%	0.1%

Eligible singleton births include singleton births with a gestational age of 37-44 weeks and information for birth weight, sex, date of birth, maternal educational attainment, parity, and a maternal age of 9 to 49 years old. Study sample includes eligible singleton births within 10 km of an air monitor active throughout pregnancy.

**Table 2:** Distribution of pollutant exposures averaged over length of pregnancy, as measured within 10 km of mother's residential geocode.

pollutant	unit	N	mean	SD	interquartile range
CO	ppm	2,853,245	0.87	0.45	0.56 - 1.09
NO_2_	pphm	2,808,662	2.42	0.95	1.69 - 3.12
O_3_	pphm	3,303,834	2.35	0.65	1.89 - 2.74
SO_2_	ppb	1,167,449	2.10	1.08	1.25 - 2.84
PM_10_	*μ*g/m^3^	1,778,579	31.4	11.2	22.6 - 38.7
PM_2.5_	*μ*g/m^3^	1,402,622	16.7	5.5	12.0 - 21.0
PM_coarse_	*μ*g/m^3^	740,885	15.7	7.5	11.0 - 18.1

In multivariate models, lower birth weight was associated with shorter gestational age, female infant sex, Black, Asian, and Hispanic mothers, younger maternal age, lower maternal educational attainment, lower parity, less access to prenatal care, being unmarried, living in neighborhoods with lower educational attainment, lower home ownership rates, and higher rates of poverty and unemployment (see Additional File 1: Multivariate modeling results for difference in birth weight for selected non-pollution variables). Pollution models were adjusted for all of these maternal, infant, and neighborhood risk factors as well as type of assigned geocode (i.e. 2000 tract, 1990 tract, or 2000 ZCTA) and calendar year and season of birth.

Table 3 shows multivariate modeling results for differences in birth weight associated with air pollution exposures for different radii distance from an air monitor. NO_2_, O_3_, PM_10_, PM_2.5 _and coarse PM were consistently linked to lower birth weight within all three different distance limits and CO was linked to lower birth weight within 5 and 10 kilometer distance limits in the linear models. NO_2 _was associated with increased odds of low birth weight across the three distance limits and CO and PM_2.5 _were associated with lower birth weight risks at the higher distance limits in the logistic models. SO_2 _was linked to higher birth weights within 5 and 10 km distance limits in the linear model, but only within 10 km in the logistic model. The associations between birth weight and the trimester-level exposures to air pollutants were similar to that between full pregnancy pollutant exposures and birth weight, although trimester effects were reversed or attenuated for some pollutants, such as CO, NO_2_, PM_10_, and coarse PM during the second trimester (Table 4). Overall, the birth weight differences were slightly stronger for the full pregnancy exposures.

**Table 3:** Multivariate model results for change in birth weight associated with full pregnancy pollutant exposures measured at 3 km, 5 km, and 10 km monitor distance.

	change in birth weight, in grams (95% confidence limits)	odds ratio of birth weight under 2,500 g (95% confidence limits)
CO, per ppm		
at 3 km	-2.5 (-5.4, 0.3)	1.02 (0.98, 1.07)
at 5 km	-5.9 (-7.8, -3.9)	1.06 (1.03, 1.09)
at 10 km	-5.4 (-6.8, -4.1)	1.04 (1.02, 1.06)
		
NO_2_, per pphm		
at 3 km	-8.3 (-9.6, -7.0)	1.03 (1.01, 1.05)
at 5 km	-9.7 (-10.6, -8.8)	1.04 (1.03, 1.05)
at 10 km	-9.0 (-9.6, -8.4)	1.03 (1.02, 1.04)
		
O_3_, per pphm		
at 3 km	-8.9 (-10.6, -7.1)	1.01 (0.98, 1.03)
at 5 km	-7.0 (-8.2, -5.8)	0.98 (0.97, 1.00)
at 10 km	-5.7 (-6.6. -4.9)	0.98 (0.97, 0.99)
		
SO_2_, per ppb		
at 3 km	1.7 (-0.3, 3.8)	1.02 (0.99, 1.06)
at 5 km	2.4 (1.0, 3.7)	1.01 (0.99, 1.03)
at 10 km	3.1 (2.3, 3.8)	1.01 (1.00, 1.02)
		
PM_10_, per 10 *μ*g/m^3^		
at 3 km	-5.5 (-6.9, -4.1)	1.00 (0.97, 1.02)
at 5 km	-7.6 (-8.5, -6.7)	1.00 (0.98, 1.01)
at 10 km	-7.2 (-7.9, -6.6)	1.00 (0.99, 1.01)
		
PM_2.5_, per 10 *μ*g/m^3^		
at 3 km	-9.2 (-12.5,-5.9)	1.04 (0.99, 1.09)
at 5 km	-11.4 (-13.5, -9.3)	1.05 (1.02, 1.08)
at 10 km	-12.8 (-14.3, -11.3)	1.04 (1.02, 1.07)
		
PM_coarse_, per 10 *μ*g/m^3^		
at 3 km	-9.4 (-12.8, -6.0)	1.00 (0.95, 1.05)
at 5 km	-10.1 (-12.2, -8.0)	0.99 (0.96, 1.02)
at 10 km	-9.3 (-10.7, -7.9)	0.99 (0.97, 1.01)

Models adjusted for infant sex, gestational age, season and year of birth, parity, maternal factors (race/ethnicity, educational attainment, marital status, access to prenatal care, birth place, age) and neighborhood SES measures (poverty rate, home ownership, educational attainment, unemployment rate).

**Table 4:** Effects of trimester-specific pollutant exposures on birth weight, in grams (95% confidence interval).

	**first trimester**^**a **^**exposure**	**second trimester**^**a **^**exposure**	**third trimester**^**a **^**exposure**
CO, per ppm			
at 3 km	-2.2 (-5.0, 0.7)	5.3 (1.7, 8.8)	-6.7 (-9.8, -3.6)
at 5 km	-2.4 (-4.4, -0.4)	3.2 (0.8, 5.6)	-7.7 (-9.8, -5.6)
at 10 km	-1.9 (-3.3, -0.6)	2.5 (0.9, 4.2)	-7.0 (-8.4, -5.5)
			
NO_2_, per pphm			
at 3 km	-2.4 (-4.4, -0.5)	1.8 (-0.8, 4.3)	-8.1 (-10.2,-6.1)
at 5 km	-3.1 (-4.4, -1.8)	0.9 (-0.8, 2.5)	-7.9 (-9.2, -6.5)
at 10 km	-3.0 (-3.9, -2.1)	0.6 (-0.6, 1.7)	-7.0 (-7.9, -6.0)
			
O_3_, per pphm			
at 3 km	-2.9 (-4.4, -1.5)	-3.1 (-4.6, -1.6)	-3.0 (-4.4, -1.5)
at 5 km	-2.7 (-3.7, -1.7)	-2.2 (-3.2, -1.1)	-2.4 (-3.4, -1.4)
at 10 km	-2.1 (-2.9, -1.4)	-2.3 (-3.1, -1.5)	-1.3 (-2.1, -0.6)
			
SO_2_, per ppb			
at 3 km	0.8 (-1.8, 3.3)	0.4 (-2.7, 3.5)	0.6 (-1.9, 3.2)
at 5 km	1.8 (0.3, 3.4)	0.1 (-1.7, 2.0)	0.4 (-1.1, 2.0)
at 10 km	2.5 (1.6, 3.4)	-0.1 (-1.1, 0.9)	0.7 (-0.2, 1.5)
			
PM_10_, per 10 *μ*g/m3			
at 3 km	-2.6 (-4.3, -0.9)	-0.3 (-2.2, 1.6)	-3.1 (-4.8, -1.3)
at 5 km	-2.7 (-3.8, -1.7)	-1.1 (-2.3, 0.1)	-4.1 (-5.2, -3.0)
at 10 km	-2.3 (-3.0, -1.6)	-1.5 (-2.3, -0.7)	-3.7 (-4.4, -3.0)
			
PM_2.5_, per 10 *μ*g/m3			
at 3 km	-6.9 (-9.6, -4.2)	-0.5 (-3.6, 2.6)	-2.4 (-5.2, 0.4)
at 5 km	-6.1 (-7.8, -4.3)	-2.2 (-4.2, -0.3)	-3.6 (-5.5, -1.8)
at 10 km	-6.0 (-7.3, -4.8)	-2.6 (-4.0, -1.3)	-4.7 (-6.0, -3.5)
			
coarse PM, per 10 *μ*g/m3			
at 3 km	-3.5 (-7.1, 0.0)	0.3 (-3.5, 4.1)	-6.7 (-10.1,-3.3)
at 5 km	-4.2 (-6.3, -2.0)	-1.2 (-3.6, 1.1)	-5.0 (-7.1, -2.9)
at 10 km	-3.4 (-4.9, -2.0)	-1.0 (-2.5, 0.5)	-5.1 (-6.4, -3.8)

^a^first trimester: first 16 weeks after last menstrual period, second trimester: weeks 17 to 28, third trimester: week 29 to delivery.

Models adjusted for infant sex, gestational age, season and year of birth, parity, maternal factors (race/ethnicity, educational attainment, marital status, access to prenatal care, birth place, age) and neighborhood SES measures (poverty rate, home ownership, educational attainment, unemployment rate)

**Figure 1:**
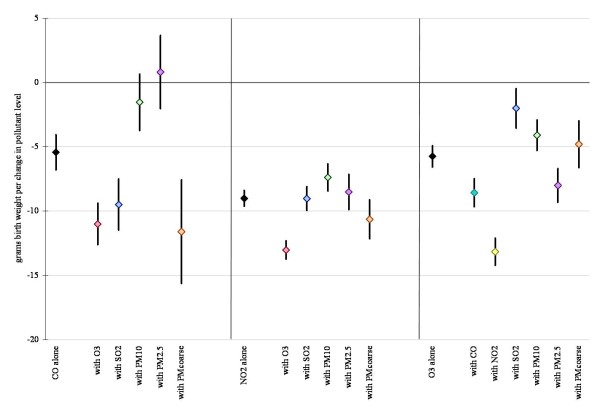
**Difference in birth weight in grams associated with full pregnancy gaseous pollutant exposures for births within 10 km monitor distance, single and two-pollutant linear models (95% confidence interval)**. Results displayed in the figures are controlled for infant's sex, gestational age, calendar year of birth, season, maternal educational attainment, age, marital status, race/ethnicity, country of birth and parity, adequacy of prenatal care, an indicator variable reflecting common medical risk factors, and neighborhood poverty rate, owner occupancy, low education rate, and unemployment rate.

**Figure 2:**
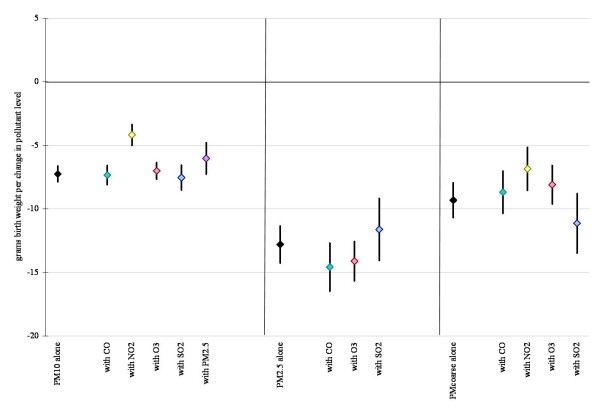
**Difference in birth weight in grams associated with full pregnancy particulate matter exposures for births within 10 km monitor distance, single and two-pollutant linear models (95% confidence interval)**. Adjustments as in Figure 1.

Figures 1 and 2 display linear model results (within 10 km monitor distance) for each air pollutant alone, and also after co-pollutant adjustment for those pollutants with a level of correlation under 70%. Results for all pollutants considered in the multivariate analysis were robust to co-pollutant adjustment remaining statistically significant in all cases, except for CO where effect estimates became insignificant with the addition of PM_10 _and PM_2.5_. Results were also robust across the different distance limits (data not shown).

**Figure 3:**
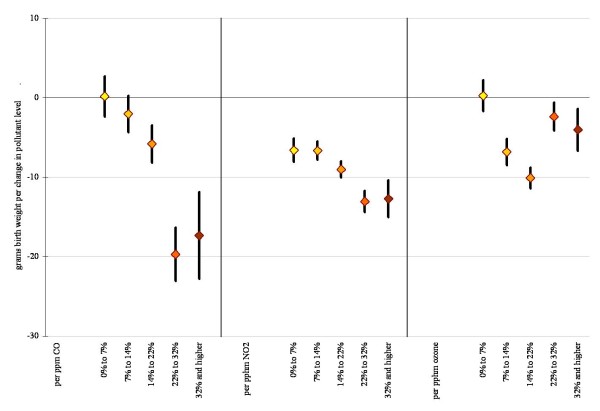
**Difference in birth weight in grams associated with full pregnancy gaseous pollutant exposures for births within 10 km monitor distance, stratified by neighborhood level poverty rate (95% confidence interval)**. Adjustments as in Figure 1.

**Figure 4:**
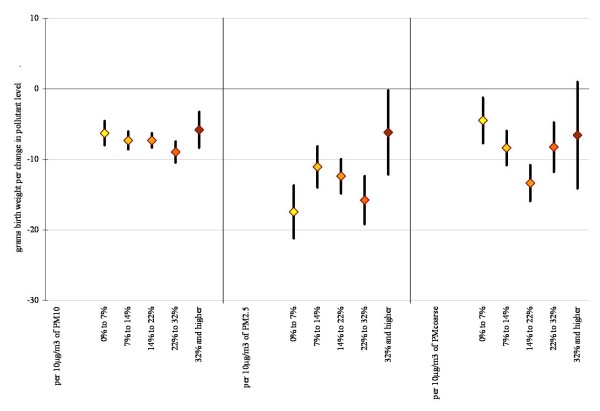
**Difference in birth weight in grams associated with full pregnancy particulate matter exposures for births within 10 km monitor distance, stratified by neighborhood level poverty rate (95% confidence interval)**. Adjustments as in Figure 1.

**Figure 5:**
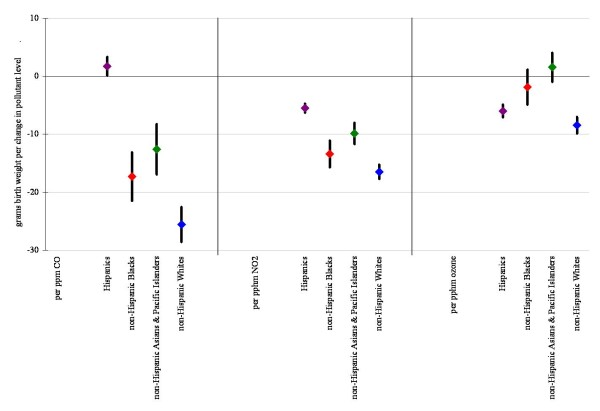
**Difference in birth weight in grams associated with full pregnancy gaseous pollutant exposures for births within 10 km monitor distance, stratified by maternal race and ethnicity (95% confidence interval)**. Adjustments as in Figure 1.

**Figure 6:**
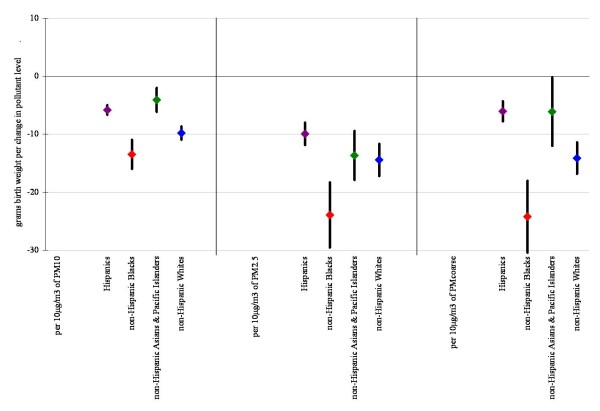
**Difference in birth weight in grams associated with full pregnancy particulate matter exposures for births within 10 km monitor distance, stratified by maternal race and ethnicity (95% confidence interval)**. Adjustments as in Figure 1.

Based on previous studies we assessed for interactions by race (Figures 3 and 4) and neighborhood level poverty rate (Figures 5 and 6). We did not find consistent evidence of effect modification by area-level poverty, although results indicated effect modification by neighborhood poverty levels for NO_2 _and CO (Figure 3). When we stratified our analysis by maternal race, results showed stronger effect estimates for Whites for some of the gaseous pollutants. However, PM_2.5 _and coarse PM effect estimates for decreases in average birth weight were strongest for African Americans (Figure 6).

## Discussion

Consistent with prior literature, we have shown a modest relationship between ambient air pollutant exposure (PM_2.5_, PM_10_, coarse PM, CO, NO_2 _and O_3_) and birth weight among full-term infants. This association between increasing pollutant exposures and decrements in birth weight persisted during different trimesters of exposure, although the strongest effects were seen for exposures during the entire gestational period. Our study results are consistent with previous studies in California which found adverse birth weight effects for PM_2.5 _[28-30,48,49], CO [26,28,30], and ozone [30] although the timing of these effects varied in terms of trimester-specific or full gestational exposure. Although smaller particles have been the focus of regulatory and scientific attention for its impacts on health [50], results from this study confirm recent work indicating that exposure to coarse particles may adversely affect birth weight [49]. Results for NO_2 _and PM_10 _also confirm previous study results in other areas, such as New England [31,38]. Although prior studies have found evidence for differential effects of air pollution among different socioeconomic groups, such as maternal race [31] or neighborhood SES [51], our results did not yield consistent evidence of interaction when we examined effect modification by neighborhood level poverty rate. However, our analysis did show stronger effect estimates for decreased average birth weight among Whites associated with some of the gaseous pollutants, while effects estimates were strongest among African Americans for PM_2.5 _and coarse PM. Our future work will re-examine potential effect modification of air pollution birth outcome relationships by individual and area-level SES factors in a larger population that includes births from several states with a broader range of pollutant burdens and neighborhood conditions.

Although we were able to control for many individual and area level factors, maternal smoking is not reported on most California birth records. Its inclusion in our study may have changed our results, had that information been available. The prevalence of cigarette smoking among pregnant women in California was 8.7% in 2003 [52] and its effects on birth weight are well documented [53]. However, recent studies suggest that although smoking during pregnancy has a large effect on birth weight, in studies of ambient air pollution it does not significantly confound the association between ambient air pollution exposure and adverse perinatal outcomes such as infant mortality and preterm birth [54,55]. Another analysis examining the effect of maternal smoking on the association between particulate matter and birth weight using birth records from Arizona and Florida found minimal changes in the effect estimates for particulate matter exposure and infant birth weight after controlling for maternal smoking [56].

The negative effects on birth weight except CO remained robust to inclusion of other pollutants, although highly correlated pollutants were not included in these models. For example, PM_10_, PM_2.5_, and NO_2 _were found to be highly correlated as well as CO and NO_2 _and tend to come from common sources. Thus, this analysis cannot assess whether those pollutants linked to lower birth weight could in fact be proxies for other pollutants with similar emission sources. Future work could deploy methods that better distinguish key common source pollutants that exert adverse effects on low birth weight. However, this single pollutant approach would not take into account the cumulative impact of exposures to multiple air pollutants, which may be important if in fact chemical mixtures lead to higher health risks than individual chemical constituents. A major source of both gaseous and particulate air pollutants is combustion, and one important area of future inquiry is to take a source-based approach to assessing health effects rather than isolating the impacts of individual pollutants. More can be done to analyze and develop source-specific measures, such as traffic density [51,57], that could elucidate opportunities for exposure reduction to multiple pollutants [24].

We assessed the consistency of our results by using different distance limits for the births we examined (3, 5 and 10 kilometers). Results for our pollutants remained statistically significant in the linear models and results varied more for the logistic models. Other studies have sought to examine the impact of exposure assessment methods on effect estimates of air pollution impacts on health outcomes. For example, a Los Angeles study demonstrated how within-city gradients of PM_2.5 _exposures produced larger effect estimates for mortality than models comparing the impact of PM_2.5 _across communities [58]. This issue has also been examined in relation to perinatal outcomes in a California study that found that the use of different air pollution exposure metrics (e.g. county-wide average, nearest monitor, distance-weighted average of monitors <5 miles of mother's residence) affected estimates for air pollution effects on birth weight [48], with greater associations between birth weight and PM_2.5 _exposures were averaged over counties rather than using monitors closer to a mother's residence. The reasons for this difference remain unclear, however. Nevertheless, these studies suggest that air pollution exposures can vary considerably at smaller scales and that this variation can affect the size of effect estimates. Efforts to further examine whether and how exposure assessment at smaller scales affect observed relationships between air pollution and perinatal outcomes is needed.

Although we sought to examine this issue by estimating pollutant effects within different distance limits to monitors, we were limited to the tract and ZIP Code-levels which prohibited finer scale assessments of geographical variations in exposure. We averaged weekly exposure estimates to derive trimester-specific and full gestation exposures, so our analysis does not account for differences in the distribution of exposures during the course of a pregnancy, or the trimester-specific exposure averages. The averaging procedure used to derive exposure measures would not reflect short-term exposures to transient spikes in air pollutant levels. We used ambient monitoring as a surrogate for personal exposure during the course of pregnancy, which does not account for indoor pollutant levels, occupational exposures, transportation-associated exposures, or other activities not occurring in one's home neighborhood. Such measurement error in exposure could have unpredictable impacts on our estimate of the effect of air pollutant exposures on birth weight. Additionally, birth records only record maternal address at the time of delivery, so we could not account for residential mobility during pregnancy. Studies vary in their estimates of how important the impact of residential mobility may be on effect estimates of air pollution on birth outcomes [24]. Any misclassification due to this trend is likely to be larger during the earlier stages of pregnancy than during the time period closer to delivery.

The majority of air pollution and birth outcome studies have focused on air pollutants that are routinely monitored and regulated with national standards, yet there are other pollutants, such as air toxics, that may also be of interest due to their respiratory, reproductive and developmental effects [59]. There is only sparse monitoring data available for air toxics, although modeled annual average estimates are now available for several periods [60]. Future studies should include impacts from other categories of pollutants that may exert harm during pregnancy.

## Conclusions

This study indicates that maternal exposure to air pollution may result in modestly lower infant birth weight. Although the effects are smaller than many other exposures, such as smoking, the ubiquity of air pollution exposures, the responsiveness of pollutant levels to planning and regulation efforts, and the fact that the highest pollution levels in California are lower than those regularly experienced in other countries suggests the potential implications may be important for infant health and development.

## Abbreviations

AQS: Environmental Protection Agency's Air Quality System; CALAIRS: California Environmental Protection Agency's Aerometric Information Reporting System; CO: carbon monoxide; LBW: low birth weight; NO_2_: nitrogen dioxide; NO_x_: nitrogen oxides; O_3_: ozone; PM: particulate matter; PM_10_: particulate matter under 10 μm in diameter; PM_2.5_: particulate matter under 2.5 μm in diameter; PPM: parts per million; PPHM: parts per hundred million; PPB: parts per billion; SO_2_: sulfur dioxide; ZCTA: ZIP Code Tabulation Area.

## Competing interests

The authors declare that they have no competing interests.

## Authors' contributions

RMF, BMJ, MP and JLS originated the research to explore the effects of air pollution during pregnancy. RMF and BMJ conceived, designed, and implemented this study; RMF led the writing and oversaw the analytical work. BMJ conducted all of the programming and statistical analysis and assisted with the writing. MP and JLS assisted with the writing and provided critical input into the manuscript. All authors have approved the final version.

## Supplementary Material

Additional file 1 **Multivariable modeling results for difference in birth weight for selected non-pollution variables**. Data table as described above. Click here for file
