# Supplementary figures and images for: Ambient air pollution exposure and full-term birth weight in California
Source: Environ Health. 2010 Jul 28;9:44. doi: 10.1186/1476-069X-9-44 (PMC2919523; doi:10.1186/1476-069X-9-44)

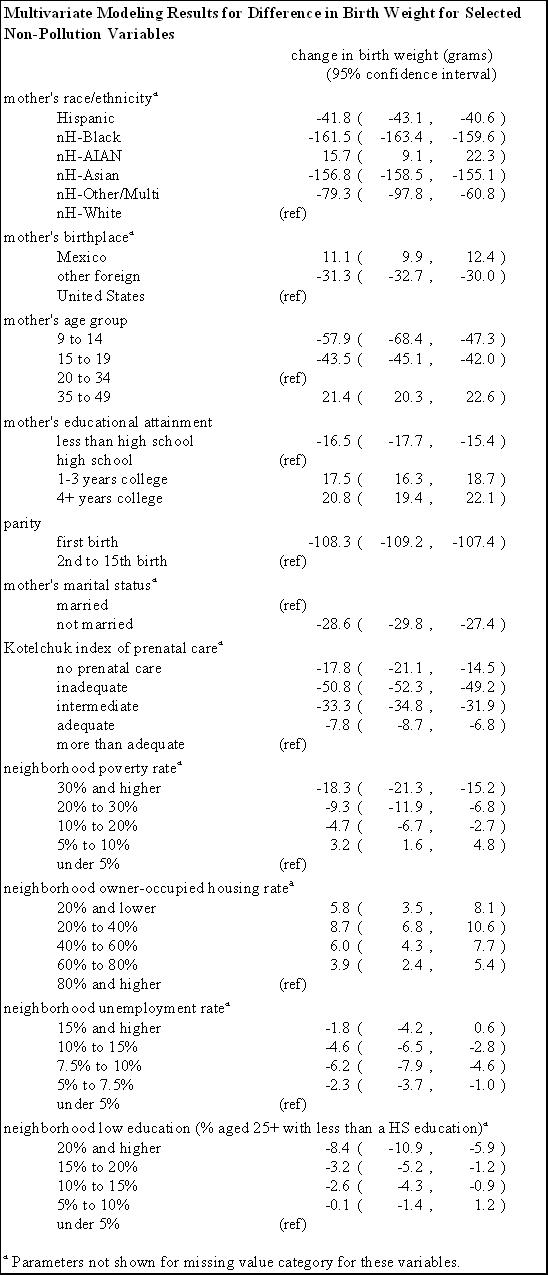

Supplement: Additional file 1 — Multivariable modeling results for difference in birth weight for selected non-pollution variables. Data table as described above. [file 1476-069X-9-44-S1.JPEG]
